# Bone Graft Paste Nanohydroxyapatite Chitosan-Gelatin (nHA/KG) for Periodontal Regeneration: Study on Three-Dimensional Cell Culture

**DOI:** 10.1055/s-0044-1800826

**Published:** 2025-03-12

**Authors:** Nadhia Anindhita Harsas, Endang Winiati Bachtiar, Lisa Rinanda Amir, Rachmat Mauludin, Sunarso Sunarso, Viona Yosefa, Maria Savvyana, Fatimah Maria Tadjoedin, Yuniarti Soeroso

**Affiliations:** 1 Department of Periodontology, Faculty of Dentistry, Universitas Indonesia, Jakarta, Indonesia; 2 Department of Oral Biology, Faculty of Dentistry, Universitas Indonesia, Jakarta, Indonesia; 3 Oral Science Research Center, Faculty of Dentistry, Universitas Indonesia, Jakarta, Indonesia; 4 Department of Pharmaceutics, School of Pharmacy, Bandung Institute of Technology, Bandung, Indonesia; 5 Department of Dental Material, Faculty of Dentistry, Universitas Indonesia, Jakarta, Indonesia; 6 Periodontology Specialist Program, Faculty of Dentistry, Universitas Indonesia, Jakarta, Indonesia

**Keywords:** periodontal regenerative therapy, nanohydroxyapatite, chitosan, gelatin, bone graft paste

## Abstract

**Objective:**

Regenerative periodontal surgical approaches require scaffolds in a form that can fill narrow and irregular defects. Each scaffold must be specially designed to conform to the shape of the specific defect. The aim of this study was to fabricate nanohydroxyapatite chitosan-gelatin (nHA/KG) pastes with different composition percentages and to analyze the differences in physical, chemical, and biological characteristics in response to periodontal tissue regeneration *in vitro* .

**Materials and Methods:**

The nHA/KG paste was prepared at three different concentrations of inorganic and organic contents (70/30; 75/25; and 80/20) by mixing nHA powder, chitosan flakes, and gelatin powder. The ratio of chitosan and gelatin on all nHA/KG pastes is 1:1. The three nHA/KG pastes were tested for the following rheology and bioactivity properties in simulated body fluid (SBF): pH value, swelling, degradability, surface morphology, and cell attachment by scanning electron microscopy and chemical structure by Fourier transform infrared (FTIR). Osteoblasts and fibroblasts were analyzed for proliferation using the MTT (3-[4,5-dimethylthiazol-2-yl]-2,5 diphenyl tetrazolium bromide) assay and for cell proliferation by reverse transcription quantitative real-time polymerase chain reaction of COL1, alkaline phosphatase (ALP), osteocalcin (OCN), and RUNX2.

**Statistical Analysis:**

Analysis of variance followed by Tukey's post hoc, Kruskal–Wallis, Wilcoxon, and paired sample *t* -tests were performed according to each data type.

**Results:**

The nHA/KG paste showed gel-like physical characteristics. The pH value after SBF immersion was stable at pH ± 7.0, although the pH of the nHA/KG 80/20 paste decreased to pH 6.3 on day 14. The three paste preparations showed significant differences in swelling ( *p* < 0.05) on days 1 and 14 and in the degradability ratio on days 1, 2, and 7 ( *p* < 0.05). The three-dimensional scaffold surface morphology differed depending on the immersion time. The FTIR test showed the presence of PO _4_^3-^ , CO _3_^2-^ , -OH, amide I, and amide II functional groups in all paste variants. The nHA/KG 75/25 paste had the most stable structure during the immersion period. Biological tests showed a viability ratio of osteoblasts and fibroblasts ≥ 70%. The paste could stimulate the messenger ribonucleic acid expression of the COL1, ALP, OCN, and RUNX2.

**Conclusion:**

The nHA/KG bone graft paste showed good potential as an injectable scaffold, with the nHA/KG 75/25 paste being the best of the three pastes tested here.

## Introduction

Damage to the periodontal ligament (PDL) tissue can lead tooth mobility and loss, thus affecting the quality of life. Therefore, the main goal for the treatment of PDL damage is to stimulate the regeneration process. One revolutionary modality that can be adapted to periodontal treatment is tissue engineering, which focuses on the replacement and regeneration of various human tissues and organs. Tissue engineering has three key components, known as the triad: cells, scaffolds, and growth factors. 1

Scaffolds in periodontal tissue engineering act as artificial matrixes that are similar to the extracellular matrix (ECM) that gives mechanical support for the growth of various cell types. 1 2 3 Scaffolds must be biocompatible, biodegradable, and have properties that stimulate osteoconduction and osteoinduction. 3 One material that resembles natural bone apatite is nanohydroxyapatite (nHA), which consists of 65% inorganic and 35% organic components. Hydroxyapatite (HA) is biocompatible, noninflammatory, nontoxic, and nonimmunogenic and easily binds to hard and soft tissue. The disadvantages of HA are that it is brittle, shows poor strength under bearing loads, and takes a long time to degrade in biological environments. 4 5

Other natural polymers that have attracted attention in biomedical applications include chitosan (K) and gelatin (G), by virtue of their biodegradability, biocompatibility, and regenerative properties. 6 7 Gelatin has the arginine-glycine-aspartate sequence also found in the ECM; therefore, it can stimulate initial cell attachment and increase cell spreading. 8 9 A combination of polymers and bioceramics (such as HA) can consequently increase bioactivity, mechanical properties, and provide HA deposition areas. This combination also offers cellular anchorage points that enable integration with the surrounding bone and regulate cell differentiation, making these materials useful in the treatment of PDL damage. 10 11

The prognosis and success of periodontal regeneration can be improved using regenerative surgical approaches that incorporate minimally invasive principles. 12 13 The bone grafts that are currently available in the market are preformed scaffolds in the form of granules, powders, and block. Meanwhile, an injectable bone graft that can change into scaffold *in situ* can accommodate the needs to fill irregular and narrow bone defects with minimal invasive surgical technique. 13 14 Testing using simulated body fluid (SBF) is recognized as the best method for studying the bioactivity of these types of materials, and current technological developments enable the creation of a natural PDL environment surrounded by ECM resembling the natural condition. 15 16 17 This three-dimensional *in vitro* model is also able to replace *in vivo* tests. 17 18

An initial SBF immersion test on bone graft paste consisting of nHA-gelatin (nHA/G) showed that nHA/G with a higher nHA content had superior degradability, pH value, calcium-to-phosphorus ratio, surface morphology, and chemical structure. The nHA/G paste also showed nontoxic properties in osteoblasts culture. Previous research also showed that 0.25% injectable HA chitosan paste supported the greatest cell viability and alkaline phosphatase (ALP) secretion for osteoblasts and was effective at inducing osteogenesis of femoral defects in rabbits. 19 20 The present study aimed to determine the best composition of nHA/KG paste that could be a scaffold *in situ* with good physical properties, biocompatible, and support the bioactivity of periodontal cells. The testing method in this study was maintained as close as possible to the protocols used for the clinical application of bone graft paste. The development of three-dimensional *in vitro* study models is expected to provide novel findings regarding cell bioactivity under conditions that resemble the natural oral environment.

## Materials and Methods

### Materials

nHA powder and chitosan flakes were obtained from the National Research and Innovation Agency (Jakarta, Indonesia). The nHA powder was prepared using a precipitation reaction involving calcium hydroxide (Ca(OH) _2_ ) and phosphoric acid (H _3_ PO) _4_ . 21 The chitosan (K) flakes were obtained from chitin extracted from shrimp shells. The gelatin (G) powder was purchased from Merck, Germany.

### Bone Graft Paste Formulation

The K flakes were dissolved in 1% acetic acid for 5 hours. The G solution was made by dissolving the powder in distilled water for 1.5 hours at 40°C. The pastes were then formed by mixing the inorganic nHA powder and the organic G and K solutions to yield ratios of nHA/KG paste of 70/30, 75/25, and 80/20 ( Table 1 ).

**Table 1:** Composition of the nHA/KG bone graft paste

nHA/KG bone graft paste	nHA	Chitosan (K)	Gelatin (G)	% Solid content	% Inorganic	% Organic
70/30	4.5 g	1 g	1 g	13	70	30
75/25	6 g	1 g	1 g	16	75	25
80/20	7.5 g	1 g	1 g	21	80	20

Abbreviation: nHA/KG, nanohydroxyapatite chitosan-gelatin.

### Rheological Tests

This study used two rheological parameters: oscillation frequency and dynamic viscosity. The oscillation frequency test was set at a strain of 0.5% and angular frequencies of 1 to 100 rad/s. Dynamic viscosity was tested using the flow sweep mode at a temperature 25°C, a sheer rate of 0.001 to 50, and a soak time of 30 seconds.

### pH, Swelling, and Degradability Tests

The SBF was prepared following the Kokubo formulation with pH of 7.4 at a temperature of 37°C. The SBF has an ion concentration similar to blood plasma in the body. 22 The bone graft paste sample (± 30 mg, weighed on an analytical balance) was placed on a glass coverslip and reweighed every 15 minutes until the weight showed no further changes; this weight was taken as the initial dry weight (W _0_ ). The pH values were first measured 5 minutes after the paste was immersed in SBF and again after 1, 2, 7, and 14 days of incubation. For the swelling test, the paste samples were weighed again at each incubation period to determine the weight post-SBF immersion (W _t_ ).

The swelling ratio was then calculated as:



After weighing for the swelling ratio, the paste samples were dried in a desiccator for 24 hours to obtain the final dry weight (W _d_ ). The degradability ratio then calculated as:



### Scanning Electron Microscopy

The desiccator-dried nHA/KG paste samples were analyzed by scanning electron microscopy (SEM) (20 kV high vacuum SEM FEI Quanta 650 ESEM) at a scale of 30 μm to evaluate the surface morphology. All samples were sputter-coated (low vacuum) with gold as an electrically conducting material. The SEM observations were performed at 150×, 1,000×, and 5,000× magnification to determine the number of pores, pore diameter, and distance between pores. The SEM test result images in .JPG format were analyzed using the ImageJ program version 1.54d.

### Fourier Transform Infrared Spectroscopy

The Fourier transform infrared (FTIR) spectrum is used to detect a structural integration and functional group present in the composites. The nHA/KG paste samples (preimmersion, and days 1, 2, 7, 14, and 28 post-SBF immersion) were tested as KBr pellets using an FTIR Nicolet iS50 instrument at a wavelength range of 4,000-400 cm ^−1^ to record the spectra. The FTIR results were analyzed using OriginPro2023 software.

### Cell Culture, MTT Assays, and Reverse Transcription Quantitative Real-Time Polymerase Chain Reaction

The MC3T3-E1 and MG-63 osteoblast and MRC-5 fibroblast cell lines were cultured in complete culture media containing Dulbecco's modified Eagle medium with 10% fetal bovine serum in T75-flasks. After reaching confluency for cell viability, the MC3T3-E1 cells were incubated with the bone graft paste for 24, 48, and 72 hours. Cell viability was determined using the MTT (3-[4,5-dimethylthiazol-2-yl]-2,5 diphenyl tetrazolium bromide) assay by spectrophotometric measurement of the optical density at 600 nm.

To evaluate osteoblastic and fibroblastic differentiation, MG-63 and MRC-5 cells were cultured using a three-dimensional cell culture technique in 24-well plates. The nHA/KG paste was placed on the bottom of each well, covered with complete medium, and cultured for 1 hour. The medium was discarded and the cells were seeded directly onto the paste surface at a cell density of 5 × 10 ^5^ cells/well. After incubation for 1 hour, complete medium was added to fill the total well volume. Both cell types were cultured for 24, 48, and 72 hours. Reverse transcription quantitative real-time polymerase chain reaction (RT-qPCR) was performed to measure the messenger ribonucleic acid (mRNA) expression of the osteoblasts and fibroblasts. The gene expression of COL-1 was tested in the MRC-5 fibroblasts, whereas ALP, osteocalcin (OCN), and RUNX2 were analyzed in the MG-63 osteoblasts with glyceraldehyde 3-phosphate dehydrogenase (GAPDH) as the housekeeping gene. The primer sequence that were used are: COL-1 forward 5′-TCTAGACATGTTCAGCTTTGTGGAC-3′ and reverse 5′ TCTGTACGCAGGTGATTGGTG-3′, ALP forward 5′-GGACCATTCCCACGTCTTCAC-3′ and reverse 5′-CCTTGTAGCCAGGCCCATTG-3′, OCN forward 5′- AGCCACCGAGAC ACCATGAGAG-3′ and reverse 5′-GTGCCTGGAGAGGAGCAGAACT-3′, RUNX2 forward 5′- GTGGACGAGGCAAGAGTT-3′ and reverse 5′-GGTGCAGAGTTCAGGGAG-3′, and GAPDH forward 5′-GAAGGTCGGAGTCAACGG-3′ and reverse 5′-GGAAGATGGTGATGGGATT-3′. 23 24 The MTT assays and RT-qPCR analyses were conducted in triplicate.

### Cell Attachment

A qualitative test was used to observe the cell morphology when the cells had attached to the nHA/KG paste. After incubation for 48 hours on the paste surface, the MG-63 and MRC-5 were fixed using 3% glutaraldehyde and then dehydrated at room temperature in a graded ethyl alcohol series (20, 50, 70, 90, and 100%) for 10 minutes. The samples were transferred to pots laminated with aluminum foil, immersed in hexamethyl dihydrate for 5 minutes, and then dried in a fume hood for 1 hour. The samples were coated with gold and examined using the SEM FEI Inspect F50 instrument under 4,000× magnification.

### Statistical Analysis

Statistical analyses were conducted using SPSS version 29. Data with normal distributions were analyzed using one-way analysis of variance followed by Tukey's post hoc test. Data without a normal distribution were analyzed using the Kruskal–Wallis test. The Wilcoxon and paired sample *t* -tests were used to analyze the changes in mRNA expression.

## Results

### Rheology

**Fig. 1:**
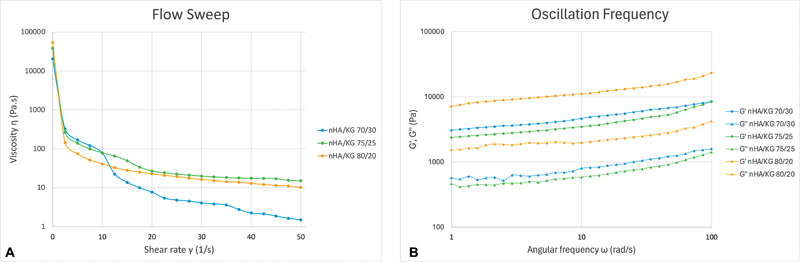
Flow sweep and oscillation frequency graphs for the nanohydroxyapatite chitosan-gelatin (nHA/KG) pastes.

The three nHA/KG pastes showed a nonlinear relationship between viscosity and sheer rate. The viscosity decreased as the sheer rate increased. All nHA/KG pastes showed values of G” > G', with both values remaining parallel throughout the frequency range ( Fig. 1 ).

### pH, Swelling, and Degradability

**Fig. 2:**
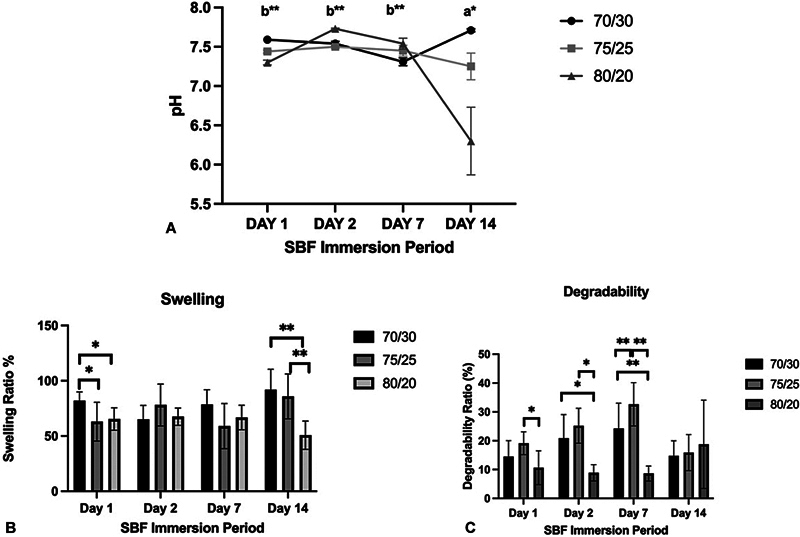
( **A** ) pH values; ( **B** ) swelling; and ( **C** ) degradability between nanohydroxyapatite chitosan-gelatin (nHA/KG) post-simulated body fluid (SBF) immersion. Analysis of variance (ANOVA), * *p* < 0.05. Kruskal–Wallis, ** *p* < 0.05.

All the nHA/KG variances showed changes in pH values between pH 7.3 and 7.7 during SBF immersion from days 1 to 14, except for nHA/KG 80/20, which showed a slight decrease to pH 6.3 on day 14 ( Fig. 2A ). On days 1 and 7 post-SBF immersion, the nHA/KG 70/30 paste had the highest swelling ratio (81.87 ± 7.88 and 78.37 ± 13.39) (95% confidence interval [CI] 88.46, 75.28 and 89.57, 67.18) and the 75/25 paste had the lowest (63.00 ± 17.50 and 59 ± 20.38) (95% CI 77.63, 48.37 and 76.05, 41.96). On day 2, the nHA/KG 75/25 paste showed the highest swelling ratio (78.12 ± 18.95) (95% CI 93.96, 62.28). The swelling ratio on day 14 showed that the increase in nHA content affected the swelling ability of the paste ( Fig. 2B ).

The degradability ratio on days 1, 2, and 7 showed the highest degradability in the nHA/KG 75/25 paste (19.13 ± 3.94, 25.20 ± 6.04, and 32.65 ± 7.50) (95% CI 22.43, 15.84, 30.25, 20.14, and 38.92, 26.37). The degradability results on day 14 differed from those determined for the other three time periods and contrasted with the swelling ratio, as the degradability ratio appeared to increase with increasing nHA content ( Fig. 2C ).

### SEM

**Fig. 3:**
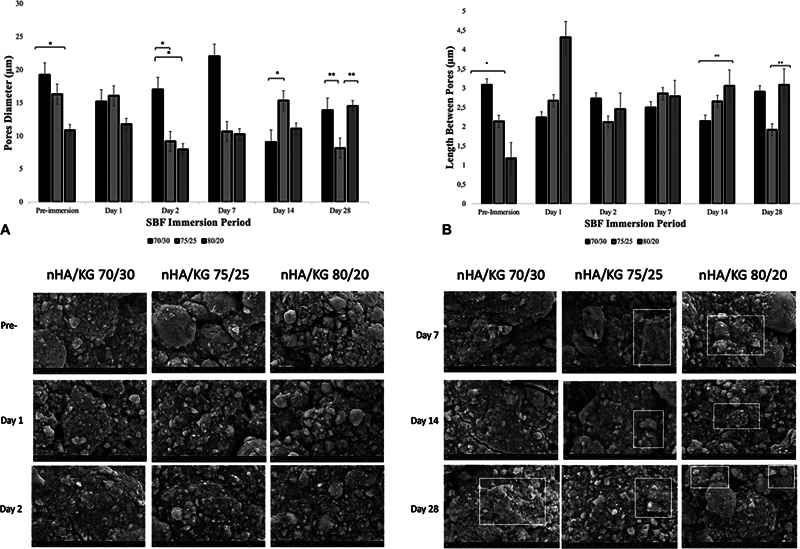
Top: Diameter ( **A** ) and length between pores ( **B** ) of nanohydroxyapatite chitosan-gelatin (nHA/KG) pastes. Preimmersion and after 1, 2, 7, and 14 days of simulated body fluid (SBF) immersion. Bottom: scanning electron microscopy (SEM) imaging with magnification 1,000× on nHA/KG pastes. Preimmersion and after 1, 2, 7, and 14 days of SBF immersion. The stratified layer is shown within white boxes. Analysis of variance (ANOVA), * *p* < 0.05; post hoc Tukey, * *p* < 0.05. Kruskal–Wallis, ** *p* < 0.05; post hoc Mann–Whitney, ** *p* < 0.05.

The nHA/KG 70/30 paste had the largest diameter at preimmersion (19.24 ± 6.88) (95% CI 24.99, 13.49), day 2 (17.03 ± 7.30) (95% CI 23.13, 10.92), and day 7 (22.05 ± 19.04) (95% CI 37.97, 6.13) post-SBF immersion. The nHA/KG 75/25 had the largest diameter at days 1 (16.07 ± 8.28) (95% CI 23.00, 9.14) and 14 (15.33 ± 6.22) (95% CI 20.53, 10.13); and the nHA/KG 80/20 had the largest diameter at day 28 post-SBF immersion (14.47 ± 3.71) (95% CI 17.57, 11.36). Measurements prior to SBF immersion showed that the nHA/KG 70/30 paste had the largest pore distance (3.09 ± 1.18) (95% CI 4.08, 2.10), and this decreased with increasing nHA content. During the SBF immersion periods, each of the nHA/KG pastes showed different changes in the lengths between pores, with statically significant differences detected at preimmersion and days 14 and 28 post-SBF immersion ( Fig. 3A, B ).

The surface morphology of nHA/KG 70/30, 75/25, and 80/20 pastes preimmersion and after SBF immersion for 1, 2, 7, 14, and 28 days, observed using SEM at 1,000× magnification, showed a general agglomeration on the surface of the nHA/KG paste, especially after SBF immersion. The nHA/KG 75/25 and 80/20 pastes showed the stratified layers after 7 days of immersion ( Fig. 3 , bottom).

### FTIR

**Fig. 4:**
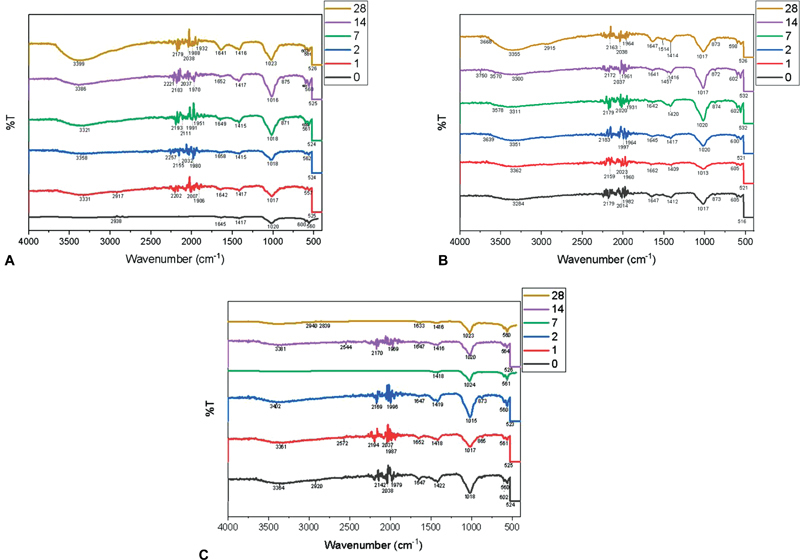
Fourier transform infrared (FTIR) of nanohydroxyapatite chitosan-gelatin (nHA/KG) pastes. ( **A** ) 70/30; ( **B** ) 75/25; and ( **C** ) 80/20. Preimmersion and at 1, 2, 7, and 14 days post-simulated body fluid (SBF) immersion.

The functional groups detected in all the three nHA/KG pastes included carbonate (1419–1409), phosphate (600–562; 1013–1024; 875–865), amide I (1662–1641), amide II (1457;1514), peptide bonds (3399–3284), and hydroxyl groups (3750–3284). No hydroxyl or amide functional groups were detected in the nHA/KG 70/30 paste prior to immersion but were increasingly detected after SBF immersion ( Fig. 4A ). In the nHA/KG 75/25 paste, all functional groups were detected during preimmersion and the wave peaks increased together with increases in the SBF immersion duration ( Fig. 4B ). The nHA/KG 80/20 paste showed a loss of some functional groups during the SBF immersion ( Fig. 4C ).

### Viability and mRNA Expression of Fibroblasts and Osteoblasts

**Fig. 5:**
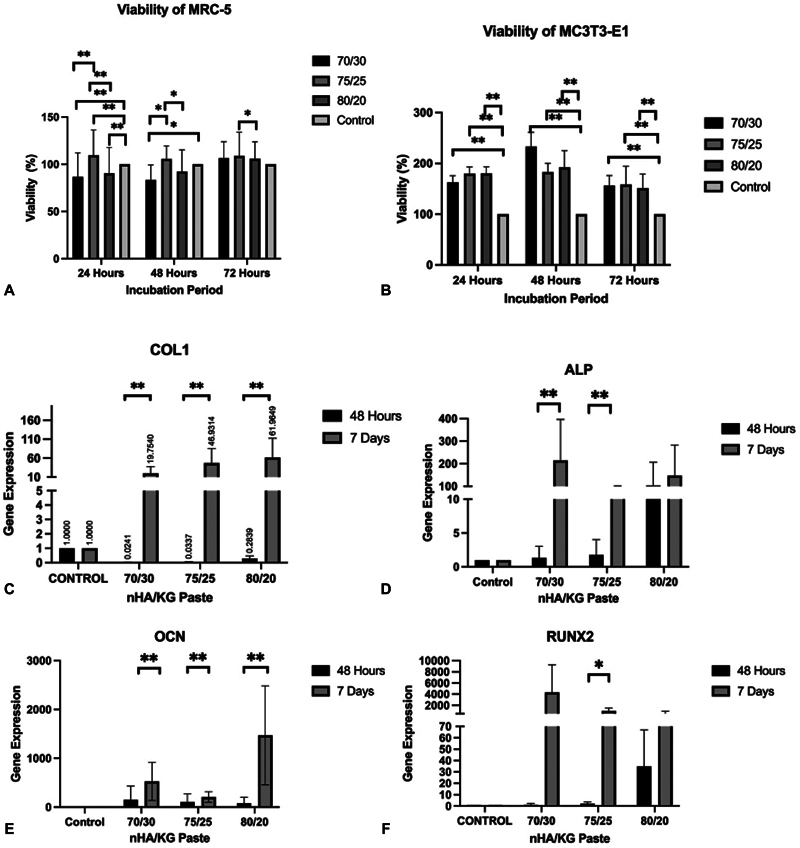
( **A** , **B** ) Viability of MRC-5 and MC3T3-E1 between nanohydroxyapatite chitosan-gelatin (nHA/KG) paste groups at 24, 48, and 72 hours. Analysis of variance (ANOVA), post hoc Tukey, * *p* < 0.05. Kruskal–Wallis, post hoc Mann–Whitney, ** *p* < 0.05. ( **C** – **F** ) COL1, alkaline phosphatase (ALP), osteocalcin (OCN), and RUNX2 gene expression of MG-63 after nHA/KG paste exposure for 48 hours or 7 days. Paired sample *t* -test, * *p* < 0.05. Wilcoxon, ** *p* < 0.05.

The MRC-5 viability after exposure to nHA/KG paste showed an average percentage of around 80 to 100%, close to the control cell viability (100%). Viability at 24, 48, and 72 hours showed significant differences ( *p* < 0.05) ( Fig. 5A ). Similar results were found for MC3T3-E1 cell viability. The cell viability was higher for all pastes at 24, 48, and 72 hours than for the control and exceeded 100% ( Fig. 5B ).

At both 48 hours and 7 days, COL1 expression increased along with the increase in nHA content in the nHA/KG paste. The COL1 gene expression across nHA/KG pastes showed an upregulation ( Fig. 5C ). The expression of the ALP, OCN, and RUNX2 genes was higher on day 7 than at 48 hours postexposure to the three nHA/KG pastes. The ALP, OCN, and RUNX2 expression values increased beginning at 48 hours ( Fig. 5D–F ), indicating the differentiation of MG-63 cells after short-term exposure to nHA/KG paste. All the three nHA/KG pastes induced upregulation of COL1, ALP, OCN, and RUNX2 gene expression.

### Cell Attachment

**Fig. 6:**
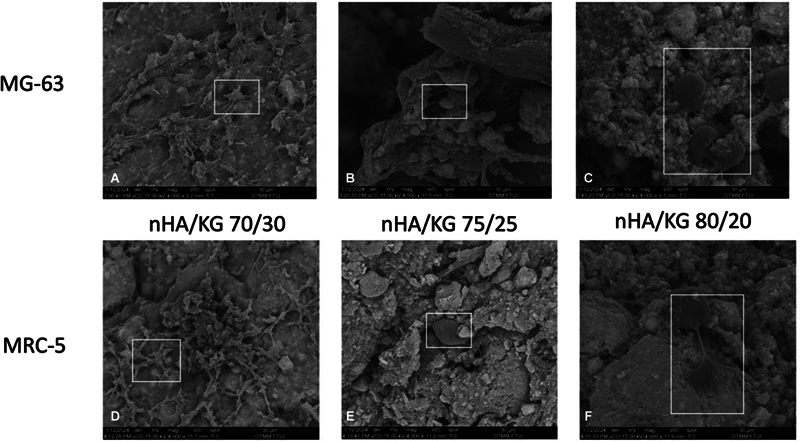
Scanning electron microscopy (SEM) results at 4,000× magnification on nanohydroxyapatite chitosan-gelatin (nHA/KG). ( **A-C** ) nHA/KG 70/30, 75/25, and 80/20 pastes, which had been cultured with MG-63 cells for 48 hours. ( **D-F** ) nHA/KG 70/30, 75/25, and 80/20 pastes, which had been cultured with MRC-5 cells for 48 hours.

It appears that all the nHA/KG paste variants can facilitate initial cell attachment at 48 hours of cell cultured, both for osteoblast and fibroblast. The morphology of MG-63 and MRC-5 cells appears to form pseudopods and adhere to the surface of the nHA/KG pastes ( Fig. 6 ). Cell distribution appeared to be spread across the graft surface.

## Discussion

The nHA/KG pastes were expected to have analogous mechanical properties with natural bone. HA is a very stiff, yet brittle material. Meanwhile, chitosan and gelatin are viscoelastic with high tensile strength but low compressive modulus. 14 25 Previous studies on injectable HA and chitosan using only hydroxypropyl methylcellulose as the emulsifier that did not have any effect on tissue regeneration. 19 20 Therefore, the combination of nHA, chitosan, and gelatin will create a more efficient composite. The addition of nHA also can hold significant osteoconductive properties. Dispersed mineral within biomaterial scaffolds can provide nucleation sites for further HA deposition and critical anchoring points that support integration with surrounding bone and regulate cellular differentiation. 11 Since the ratio of chitosan and gelatin in this study remained constant, it was possible to observe the effect on nHA in the paste more clearly.

The shape of HA used in nHA/KG is needle-like aggregation, anisotropic, and can influence viscosity. 21 26 The addition of nHA to the pastes used in this study resulted in increased stability, meaning that the paste was not easily deformed. 27 All three nHA/KG pastes appeared to have thixotropic properties, as indicated by the nonlinear relationship between viscosity and shear rate. Rheological properties are very time-dependent, leading to thixotropic features, because aggregation and breakup kinetics are not immediate. 26 Shear rate is related to the flowability when the paste is removed from the carrier, because when the syringe is depressed, the viscosity of the paste decreases, allowing it to be ejected. This property is useful when the pastes are injected inside bone defects, as the material can follow the shape of the bone defect and subsequently polymerize *in situ* . 26 The ability to use an *in situ* scaffold system shortens the operation time, while reducing the need for large tissue retractions, the size of scar tissue, and postsurgical pain. 13 All the three tested nHA/KG pastes showed comparable values of G” > G' with G' and G” throughout the frequency range. This suggests that, while they were still, all nHA/KG pastes were viscoelastic materials that looked like gels. 28 The nature of the oscillations will influence the ability of a paste to adjust its shape when stored. Study from Sohrabi et al, on a combination of bioactive glass, chitosan, and gelatin paste showed similar result of G” > G' with the nHA/KG paste. 25

In this study, a significant difference was noted between the pH of the paste after SBF immersion on days 1 to 14, with the pH range for all the three nHA/KG pastes recorded as pH 7.25 to 7.71. A pH value in this range is in accordance with research by Nur Maulida et al, who reported the pH range for HA-gelatin paste as pH 7.35 to 7.62. 29 The nHA/KG 80/20 paste after 14 days of SBF immersion showed a decrease in pH to pH 6.29 ± 0.43. A decrease in pH can occur due to the dissolution of part of the HA and because of polymer degradation. 30 31 The results for the nHA/KG 80/20 paste showed the highest degradability ratio on day 14 and many functional groups were missing according to the FTIR analysis. The pH value of SBF can affect the morphology of HA. *In situ* it is known that low pH will stimulate osteoclast function and work as a factor that regulates bone matrix production by osteoblast. Conversely, high pH (more basic and approaching alkaline pH) can increase proliferation and differentiation of osteoblast *in vitro* . 32

The nHA/KG 70/30 paste on days 1, 7, and 14 had the highest swelling ratio. This is in line with the increase in the –OH functional groups seen in the FTIR testing. In the nHA/KG 70/30 and 75/25 pastes, the numbers of –OH functional groups increased with increases in the SBF immersion period. The presence of chitosan and gelatin in a scaffold can cause swelling by absorbing water into the structure. The amide I (1662–1641) and II functional groups (1457;1514), indicating the presence of polymers in the nHA/KG paste, were clearly detected throughout the test period in the nHA/KG 75/25 paste variant, and this functional group peak increased with the duration of SBF immersion. For the other two pastes, the amide functional groups were occasionally not detected. In the nHA/KG 80/20 paste, functional groups appear to be released along with the SBF immersion time. The peak of the –OH functional group was detected in the presoaking period until days 2 and 14, but disappeared on days 7 and 28. The phosphate and carbonate functional group was highest detected in the preimmersion period, and gradually decreased over time. This can be caused because the nHA/KG 80/20 paste has the highest inorganic nHA content, thus reducing the swelling capacity and the polymer that functions to bind nHA is easily decomposed. The SBF solution content needs further analysis to confirm the ion changes that occur between the graft paste and the surrounding environment.

Swelling in scaffold can represent the water absorption ability that is related to its three-dimensional and microstructure, as well as the hydrophilicity of the molecules. The swelling process can increase pore size and porosity, thereby helping in the supply of nutrients and oxygen to the composite tissue. This ability is important for cells penetrating into composite pores during wound healing. 25 The nHA/KG paste with more HA content had a larger pore size and showed a low swelling ratio and high degradability, confirming that the polymer elements detected by FTIR will increase in size when influenced by body fluids.

Scaffold degradation occurs simultaneously with the formation of new tissue, and gaps caused by degradation are used to maintain the integrity of the implantation area during the regeneration period. 33 In this study, a dynamic change was shown to occur in the degradability ratio of the three pastes. The nHA/KG 70/30 and 75/25 pastes showed the same pattern, in which the degradability ratio continued to increase until day 7 and then decreased on day 14. The nHA/KG 80/20 paste had the highest degradability ratio on day 14, but the difference was not statistically significant when compared with the other two pastes. The FTIR results for the nHA/KG 80/20 paste showed that many functional groups disappeared as the duration of the SBF immersion period increased. This could indicate that several ions had dissolved into the environment. Li et al found that low degradation of a HA and chitosan scaffold during the initial 14 days was due to the degree of deacetylation and high molecular weight of chitosan, as well as the presence of a few intermolecular hydrogen bonds and a low lysosomal degradation ratio. 34

The SEM analysis revealed decreases in both the pore diameter and the length between pores with increasing nHA content. Wattanutchariya and Changkowchai and Rianti et al also found lower porosity values in chitosan-gelatin scaffolds with more HA content. 8 35 The pore diameter and length between pores for all the three nHA/KG pastes showed changes during SBF immersion. Differences were also evident for the surface morphology of all pastes pre- and post-SBF immersion. The stratified layers that developed during immersion can indicate apatite formation. The altered composition and surface roughness will provide chemical signals that influence cell proliferation. 36

The MTT assay was used to evaluate the change from the tetrazolium rings from MTT into formazan crystal due to the activity of mitochondria of the living cells. The insoluble formazan salt is dissolved by the addition of solubilizing agents. The color changes will be measured quantitatively as optical density values using spectroscopic multiple reader, which indicated the viability of the cells. All the three nHA/KG pastes had viability percentages above 70% for MRC-5 cells and 100% for MC3T3-E1 cells, indicating that the pastes are nontoxic. 28 The release of Ca ^2+^ and PO _4_^3-^ ions from HA, which anchors the chitosan, can produce a natural tissue-like environment for cell adhesion and proliferation. 28

The roughness and topography at the micro- and nanoscale can influence cell morphology and proliferation. Kong et al reported higher MC3T3-E1 cell viability on nHA-chitosan scaffolds than on chitosan scaffolds, even though both scaffold types were coated with apatite. 36 The metabolic ratio of PDL fibroblasts cultured on nHA paste also increased due to the phosphorylation of focal adhesion kinase. 37 Particles with irregular and sharp shapes, such as nanorods, enhance HA mechanobiology due to the inflammatory response that stimulates osteoblast adhesion, proliferation, and ALP activity when compared with round-shaped particles of the same size. 28 33 Our SEM evaluation also showed that all nHA/KG pastes could facilitate cell attachment beginning early in the culture period. In line with the findings of Maji et al, the morphology of MG-63 and MRC-5 cells appeared to form pseudopods and adhered to the surface of the graft pastes. 38 To further prove the existence of cells adhesion and morphological changes, quantitative tests need to be conducted.

The expression of the COL1 gene showed significant differences between the nHA/KG paste test groups and the controls at 48 hours and 7 days. Souto-Lopes et al also found a higher increase in collagen A1 gene expression on day 14 of treatment with nHA/chitosan scaffolds. 39 The ALP expression at 48 hours of incubation showed significant differences between groups. ALP is a marker of osteogenic differentiation that can be expressed in the early phase of osteogenesis and the pattern of upregulation of this gene indicates bone mineralization and maturation processes. 40 The OCN expression showed significances between the 70/30, 75/25, and 80/20 pastes and the control at 48 hours and day 7. 41 Previous studies reported OCN expression of human osteoblasts on chitosan/nHA scaffolds and higher OCN expression in a HA-chitosan-gelatin composite than in pure gelatin. 38 41

The RUNX2 gene has an important role in osteoblast differentiation and is related to regulatory factors involved in the expression of osteogenesis-related genes. 40 As with the other genes, RUNX2 expression was upregulated by all three paste variants, in line with previous studies that showed significantly higher upregulation of RUNX2 expression on HA-chitosan-gelatin composite scaffolds than on pure gelatin. RUNX2 expression is known to increase at the early differentiation stage of osteogenesis-derived cells and will stabilize after the initial phase is completed. 38 This pattern usually occurs during the differentiation period of preosteoblasts into mature osteoblasts. 11

The method on this study was selected as close as the clinical application, but there are still several limitations that need further analysis. The development of nHA/KG into scaffold *in situ* needs to be proven *in vivo* , so are the negative washout effect that will impair the fixation process inside a bone defect. Moreover, the viability and gene expression were only tested on the early time of regeneration process, the dynamics of the cell differentiation process until maturity were not well described. Therefore, an investigation on later period is needed. To make the nHA/KG paste easier to use, the carrier of the nHA/KG paste needs to be selected carefully.

## Conclusion

The combination of nHA powder, chitosan, and gelatin tested here showed good physical and chemical characteristics, indicating promise as injectable bone grafts. A comparison of all three pastes showed that the nHA/KG 75/25 paste has the best potential to be a scaffold *in situ* due to their physical and chemical properties during the SBF immersion period. It has a pH value of around 7, stable chemical structure, adequate swelling, and degradation ability. The responses of fibroblasts and osteoblasts also confirmed that the nHA/KG 75/25 paste is not toxic and can stimulate cell differentiation.
